# The Synthesis of NiCo_2_O_4_–MnO_2_ Core–Shell Nanowires by Electrodeposition and Its Supercapacitive Properties

**DOI:** 10.3390/nano9101398

**Published:** 2019-10-01

**Authors:** Ai-Lan Yan, Wei-Dong Wang, Wen-Qiang Chen, Xin-Chang Wang, Fu Liu, Ji-Peng Cheng

**Affiliations:** 1 College of Water Resources and Environmental Engineering, Zhejiang University of Water Resources and Electric Power, Hangzhou 310018, China; yal200@126.com; 2 State Key Laboratory of Silicon Materials, School of Materials Science and Engineering, Zhejiang University, Hangzhou 310027, China21626015@zju.edu.cn (W.-Q.C.); liufu@zju.edu.cn (F.L.); 3 Key Laboratory of Material Physics of Ministry of Education, Zhengzhou University, Zhengzhou 450052, China; wxclhm@zzu.edu.cn

**Keywords:** NiCo_2_O_4_ nanowires, MnO_2_ nanoflakes, electrodeposition, supercapacitors

## Abstract

Hierarchical composite films grown on current collectors are popularly reported to be directly used as electrodes for supercapacitors. Highly dense and conductive NiCo_2_O_4_ nanowires are ideal backbones to support guest materials. In this work, low crystalline MnO_2_ nanoflakes are electrodeposited onto the surface of NiCo_2_O_4_ nanowire films pre-coated on nickel foam. Each building block in the composite films is a NiCo_2_O_4_–MnO_2_ core–shell nanowire on conductive nickel foam. Due to the co-presence of MnO_2_ and NiCo_2_O_4_, the MnO_2_@NiCo_2_O_4_@Ni electrode exhibits higher specific capacitance and larger working voltage than the NiCo_2_O_4_@Ni electrode. It can have a high specific capacitance of 1186 F·g^−1^ at 1 A·g^−1^. When the core–shell NiCo_2_O_4_–MnO_2_ composite and activated carbon are assembled as a hybrid capacitor, it has the highest energy density of 29.6 Wh·kg^−1^ at a power density of 425 W·kg^−1^ with an operating voltage of 1.7 V. This work shows readers an easy method to synthesize composite films for energy storage.

## 1. Introduction

Energy storage is an important issue for renewable energy application. In addition to fuel cells and Li-ion batteries, supercapacitors (SCs) have drawn much attention due to their high power density, rapid charge/discharge rate, long life and easy fabrication [1,2,3,4,5]. They are even considered as an alternative to conventional batteries. However, the actual energy density of SCs is not as high as that of batteries due to their different mechanism for energy storage [6]. Therefore, many methods have been developed to enhance the energy density of SCs. Actually, the electrode material is a key factor in determining the principal performances of a SC including specific capacitance, cycling stability and rate capability, typically derived from the structural defects [7,8]. Apart from carbon-based materials, transition metal oxide/hydroxide, metal sulfides and their composites [9,10,11,12,13,14], recently, eggshell materials have been reported as electrode materials of SCs [15,16]. 

Carbon materials usually have low specific capacitance due to the electrical double-layer capacitance (EDLC) arising from the electrostatically stored surface energy at the interface of electrode materials, though they possess high specific surface areas [5,6,15,16]. Conductive polymers can have a higher specific capacitance than carbon materials, but a lower cycling stability [17]. In comparison to carbon materials and conductive polymers, transition metal oxides (TMOs) have a larger theory specific capacitance and higher electrical conductivity because of Faradaic reactions or pseudo-capacitance. During the charge and discharge processes, redox reactions occur on the material’s surface and energy transfer between electrolyte and electrode, like battery electrodes. So, diverse TMOs with different compositions and structures are synthesized for SCs in order to obtain a large energy density [18,19,20].

Recently, several strategies have been well developed to improve the performances of TMOs. One is designing hierarchical structures composed of different TMOs, for example the core–shell structured composites, in which a highly-conductive core material is coated by a highly-active shell to form synergistic effects [21,22]. Thus, a TMO material with a high conductivity and one-dimensional (1D) morphology is readily selected as the core and deposited onto current collectors to form a film at first, providing more electron transport pathways and reaction sites. Then another 2D TMO is chosen as the shell material due to its high surface area [23]. A synergistic combination of rich Faradaic reactions will be generated from each component. So, TMO composite films deposited on current collectors are commonly designed and fabricated as electrodes for SCs due to their high utilization of electrode materials. Among various TMOs, 1D nickel cobaltite (NiCo_2_O_4_) nanowires possess larger specific capacitance and higher electrical conductivity than common TMOs. 1D NiCo_2_O_4_ nanowire films grown on Ni foams are deemed as one of most potential candidates for the deposition of guest TMO materials, such as CoMoO_4_ [4], rGO [24], Co_3_O_4_ [25], NiWO_4_ [26], NiMoO_4_ [27], MnO_2_ [28,29,30,31,32,33], etc. Among different TMOs, MnO_2_ with a 2D morphology is also a popular pseudocapacitive material due to its low cost and environmental benignity, but with a low specific capacitance. In order to overcome the drawbacks of MnO_2_ and combine the advantages of NiCo_2_O_4_ nanowires, their hierarchical core–shell structures have been investigated for SCs [3,28,31,32,33,34,35,36,37].

The other strategy is building hybrid SCs, i.e., the two electrodes are different [2,38]. Though there are few commercially available hybrid SCs on the market, they are intensively studied in laboratories and deemed as potential directions for SCs. Hybrid SCs are easily classified into two kinds, EDLC//redox and redox//redox [39,40]. A high operating voltage in aqueous electrolyte can be achieved, even up to 2.0 V, thus leading to a high energy density, because E = ½ × C(ΔV)^2^. However, the stored energy density of hybrid SCs is greatly dependent on the two electrode materials. Thus, the type of EDLC//redox hybrid SCs is of great significance for practical application [2,38].

In this work, 2D MnO_2_ deposited on 1D NiCo_2_O_4_ nanowires are synthesized and investigated as electrodes of SCs. Some previous papers have reported the fabrication of NiCo_2_O_4_–MnO_2_ core–shell structures for SCs [28,31,32,33,34,35,36,37,41]. However, the most common approach is chemical deposition using strongly oxidative KMnO_4_ to deposit MnO_2_ onto NiCo_2_O_4_ [31,33,34,35,36,37]. Both the conductive substrate and the pre-coated NiCo_2_O_4_ will be affected by the chemical reaction during the generation of MnO_2_. Compared with the chemical method under severe conditions, electrochemical deposition of MnO_2_ can be carried out under much milder conditions and higher efficiency [28,32,42], showing few impacts on the conductive substrate and NiCo_2_O_4_. In this paper, NiCo_2_O_4_–MnO_2_ core–shell nanowires are thus synthesized by electrodeposition method. The NiCo_2_O_4_ nanowires are pre-deposited on the surface of nickel foam with chemical deposition and calcination. Subsequently, the electrochemical performances of NiCo_2_O_4_–MnO_2_ core–shell composite were investigated and it was combined with activated carbon (AC) to assemble hybrid SCs.

## 2. Experimental Section

### 2.1. The Synthesis of NiCo_2_O_4_–MnO_2_ Core–Shell Nanowires

**Figure 1:**
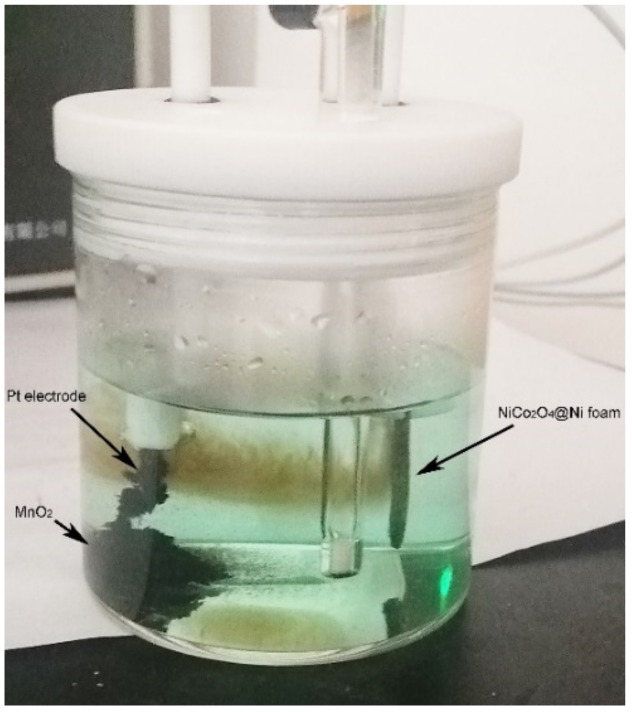
Photograph of electrodepositing MnO_2_ onto NiCo_2_O_4_@Ni.

All chemicals were analytical grad and purchased from Sinopharm Chem. Reagent Co., Ltd., Shanghai, China. The synthesis of 1D NiCo_2_O_4_ nanowire films on nickel foam as a substrate (NiCo_2_O_4_@Ni) was carried out by the hydrothermal method and calcination at 300 °C, as we previously reported [43]. The subsequent growth of 2D MnO_2_ nanoflakes on NiCo_2_O_4_ nanowires as backbones was performed by a facile electrodeposition technique. The experiment was carried out in a three-electrode glass cell, where NiCo_2_O_4_ nanowires deposited on nickel foam (1.5 cm × 1.5 cm) were used as a work electrode, a saturated calomel electrode and a Pt plate as a reference electrode and counter electrode, respectively [44]. The electrolyte was 0.02 M Mn(NO_3_)_2_ aqueous solution. A typical photograph of the electrodeposition setup is shown in Figure 1. MnO_2_ nanoflakes were deposited by the potential static with −1.0 V for 10 min. Then the nickel foam was taken out and rinsed with water and ethanol repeatedly, finally dried at 80 °C. The mass loading for materials was determined by weighing the nickel foam before and after deposition. In addition to obtaining MnO_2_@NiCo_2_O_4_ nanowires on nickel foam (MnO_2_@NiCo_2_O_4_@Ni), some MnO_2_ powder was also produced near the Pt electrode, as illustrated in Figure 1. 

### 2.2. Material Characterization

The phase of the products was measured by X-ray diffraction using an X-ray diffractometer (XRD, LabX XRD-6000, Shimadzu Ltd., Kyoto, Japan). The structure of the fabricated materials was determined by a transmission electron microscope (TEM, Philips CM200 at 160 KV, Philips Ltd., Amsterdam, Holland) and a scanning electron microscope (SEM, Hitachi-4800 at 5 KV and 8 mm, Hitachi Ltd., Tokyo, Japan). The electrochemical performances of MnO_2_@NiCo_2_O_4_@Ni and NiCo_2_O_4_@Ni were tested in a three-electrode configuration by using them as the work electrode directly, Ag/AgCl electrode and Pt plate as the reference electrode and counter electrode, respectively, in 2 M aqueous KOH. All the electrochemical properties were measured by an electrochemical analyzer (CHI 660E, Shanghai Chenhua Ltd., Shanghai, China) at room temperature. The mass loadings of NiCo_2_O_4_ and MnO_2_@NiCo_2_O_4_ nanowires were scaled to be 1.2 mg·cm^−2^ and 1.4 mg·cm^−2^ on nickel foam, respectively.

### 2.3. Hybrid Capacitor

A hybrid capacitor with MnO_2_@NiCo_2_O_4_@Ni as the positive electrode and AC@Ni as the negative electrode was assembled. The performances were measured in a two-electrode assembly in 2 M KOH as the electrolyte [43]. The specific surface area of AC is about 780 m^2^·g^−1^. The mass loadings of MnO_2_@NiCo_2_O_4_ nanowires and AC in the two electrodes were optimized beforehand to keep charge storage efficient. The optimized mass ratio of MnO_2_@NiCo_2_O_4_ nanowires to AC was about 1:2.01.

## 3. Results and Discussion

### 3.1. Structure and Chemical Analysis

**Figure 2:**
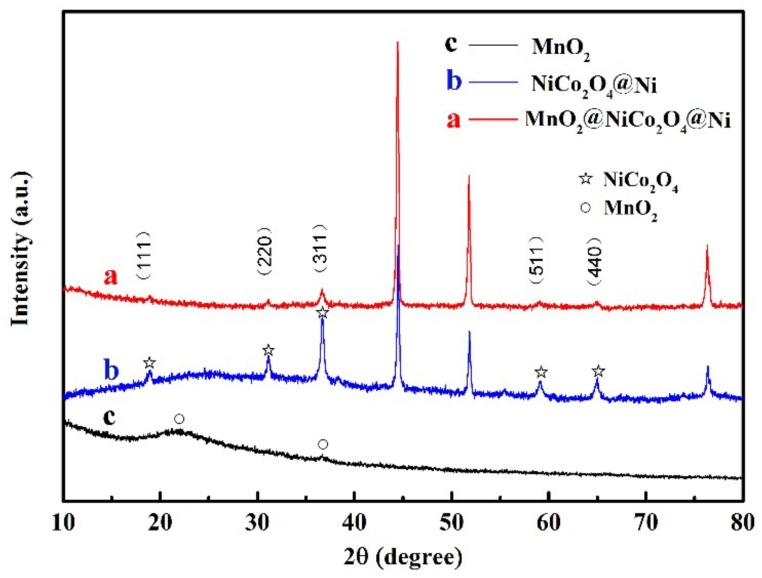
XRD patterns for the materials, (**a**) MnO_2_@NiCo_2_O_4_@Ni, (**b**) NiCo_2_O_4_@Ni and (**c**) MnO_2_ powder.

The XRD patterns of the as-prepared materials are exhibited in Figure 2. The pattern of NiCo_2_O_4_@Ni is curve b, in which the strong reflection from nickel foam at about 44.5°, 51.8° and 76.3°, and the reflection from NiCo_2_O_4_ nanowires (PDF card No. 20−0781) can be clearly seen. The corresponding planes of crystalline NiCo_2_O_4_ are marked in the figure. Our previous work proved that dense 1D NiCo_2_O_4_ nanowires were uniformly deposited on the surface of nickel foam with an average length about 5 μm to form a thin nanowire film [43]. It can be then used as a substrate to electrodeposit 2D MnO_2_ nanoflakes in this work. The XRD pattern for MnO_2_@NiCo_2_O_4_@Ni is shown as curve a in Figure 2. It shows a similar reflection pattern to NiCo_2_O_4_@Ni owing to the poor crystallinity and low mass loading of MnO_2_, about 0.2 mg·cm^−2^. However, the relative intensity ratio of the NiCo_2_O_4_ reflection to that of the Ni foam is dramatically decreased after coating with MnO_2_. It further means that the coating of MnO_2_ results in a decreased intensity of the NiCo_2_O_4_ phase. As exhibited in Figure 1, pure MnO_2_ product is also generated in the electrolyte during the deposition of MnO_2_ nanoflakes on NiCo_2_O_4_ and it is located near the Pt electrode. The XRD pattern for the powder MnO_2_ is measured and shown in Figure 2 as curve c. The weak and broadening reflection at about 23.8° and 37.3° can be indexed to (110) and (021) planes of γ-MnO_2_, respectively, indicating its low crystallinity [45,46,47].

**Figure 3:**
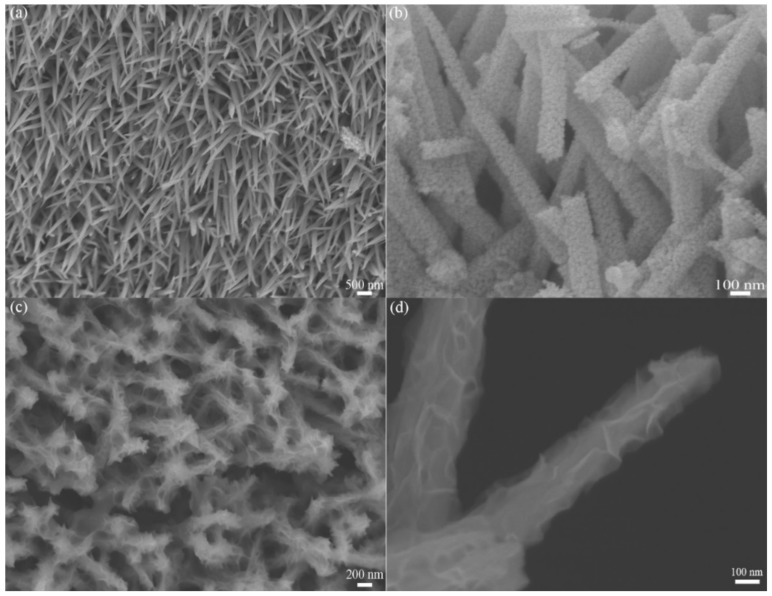
SEM images of (**a**,**b**) NiCo_2_O_4_ nanowires on Ni foam and (**c**,**d**) MnO_2_@NiCo_2_O_4_ under different magnifications.

SEM images of NiCo_2_O_4_ nanowires and MnO_2_@NiCo_2_O_4_ under different magnifications are shown in Figure 3. As shown in Figure 3a, we can see that a large number of NiCo_2_O_4_ nanowires with sharp tips are formed as a film on nickel foam. A highly magnified image in Figure 3b shows that NiCo_2_O_4_ nanowires are brittle with an average diameter of about 200 nm and made up of many smaller particles, clearly showing their porous nature. After electrodeposition of MnO_2_, NiCo_2_O_4_–MnO_2_ core–shell nanowires are then formed over the skeleton of nickel foam, as exhibited in Figure 3c. Compared with Figure 3a, the smooth surface of the NiCo_2_O_4_ nanowires is entirely coated by interconnected 2D MnO_2_ nanoflakes. Two core–shell NiCo_2_O_4_–MnO_2_ nanowires under a high magnification are shown in Figure 3d to exhibit their structures in detail, where we can see that MnO_2_ nanoflakes uniformly cover the NiCo_2_O_4_ nanowires, and they connect to each other to generate a porous morphology. Thus, from SEM results, we confirm the formation of a core–shell configuration with 1D NiCo_2_O_4_ nanowires as the core and 2D MnO_2_ nanoflakes as the shell. The heterostructure is porous with a 3D network-like structure that can provide rapid transport pathways for the enhancement of supercapacitive performances. The morphology of the MnO_2_ powder generated in the electrolyte was also characterized. Pure MnO_2_ powder was composed of spherical grains with flower-like nanosheets on their surfaces with a size about 200 nm.

**Figure 4:**
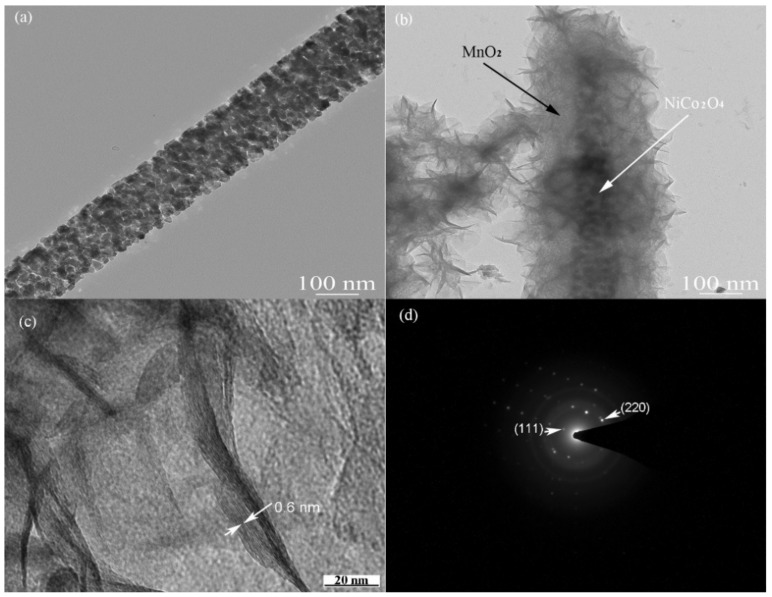
TEM images of (**a**) a NiCo_2_O_4_ nanowire, (**b**,**c**) MnO_2_@NiCo_2_O_4_ nanowires and (**d**) their electron diffraction pattern.

A typical TEM image of a straight NiCo_2_O_4_ nanowire is shown in Figure 4a. It has a diameter of about 150 nm and is comprised of a lot of small grains about 5 nm in size [43], consistent with the SEM observation. The TEM images of NiCo_2_O_4_ nanowires coated by 2D MnO_2_ flakes are exhibited in Figure 4b. The core–shell configuration of NiCo_2_O_4_–MnO_2_ has a larger diameter than the NiCo_2_O_4_ nanowire, showing that MnO_2_ nanoflakes indeed are deposited on the NiCo_2_O_4_ nanowires. These interconnected 2D MnO_2_ flakes are closely bonded to the 1D NiCo_2_O_4_ nanowire to form a porous structure. A typical high resolution TEM image in Figure 4c presents the cross-sectional image of MnO_2_ nanoflakes with a d-spacing of about 0.6 nm, belonging to the (001) crystallographic plane of birnessite-type MnO_2_ [31]. In Figure 4d, we can see the electrode diffraction pattern of a core–shell NiCo_2_O_4_–MnO_2_ nanowire. The (111) and (220) planes of cubic NiCo_2_O_4_ are pointed out in the pattern. The electron diffraction pattern derived from NiCo_2_O_4_ looks more like single crystalline, proving that these smaller NiCo_2_O_4_ particles are connected by oriented attachment. Few diffraction spots and rings from the MnO_2_ phase can be identified due to its poor crystallinity.

**Figure 5:**
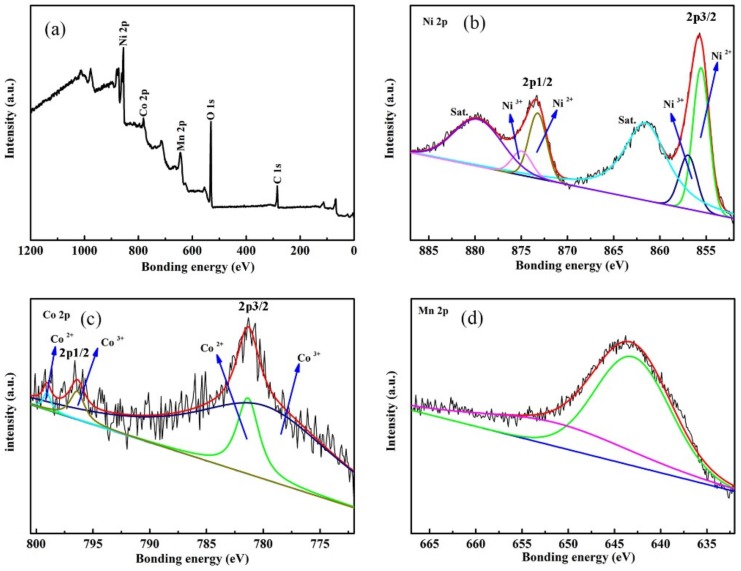
XPS spectra for core–shell NiCo_2_O_4_–MnO_2_, (**a**) wide spectrum, fine spectra of (**b**) Ni, (**c**) Co and (**d**) Mn.

The composition and valance state of core–shell NiCo_2_O_4_–MnO_2_ nanowires were determined by X–ray photoelectron spectroscopy (XPS) measurement. The XPS wide spectrum for the material is shown in Figure 5a, showing the existence of elements C, O, Mn, Co and Ni in the composite film. The fitted fine spectra of Ni 2p, Co 2p and Mn 2p are exhibited in Figure 5b–d, respectively. From the Ni 2p spectrum in Figure 5b, it has two spin-orbit doublets in 2p1/2 and 2p3/2 configurations at about 873 eV and 856 eV, respectively, together with two small satellite peaks (indicated as “Sat.”). The peaks located at about 856 eV, 857 eV, 873 eV and 875 eV prove the existence of Ni^3+^ and Ni^2+^ [43]. The two peaks for Co 2p1/2 and Co 2p3/2 at positions around 797 eV and about 781 eV, respectively, are not very strong in Figure 5c, which is caused by the wrapping of MnO_2_ films. Thus, only weak signals from Co can be detected. The peaks at about 779.5 eV and 795 eV are from Co^3+^. The other two peaks at about 781 eV and 797 eV are from Co^2+^ [43]. The binding energy separation of the Mn 2p3/2 peak at about 642.1 eV and the Mn 2p1/2 peak at 654 eV is about 11.9 eV, in good agreement with a previous report for MnO_2_ [28], as exhibited in Figure 5d. 

### 3.2. Electrochemical Measurement

**Figure 6:**
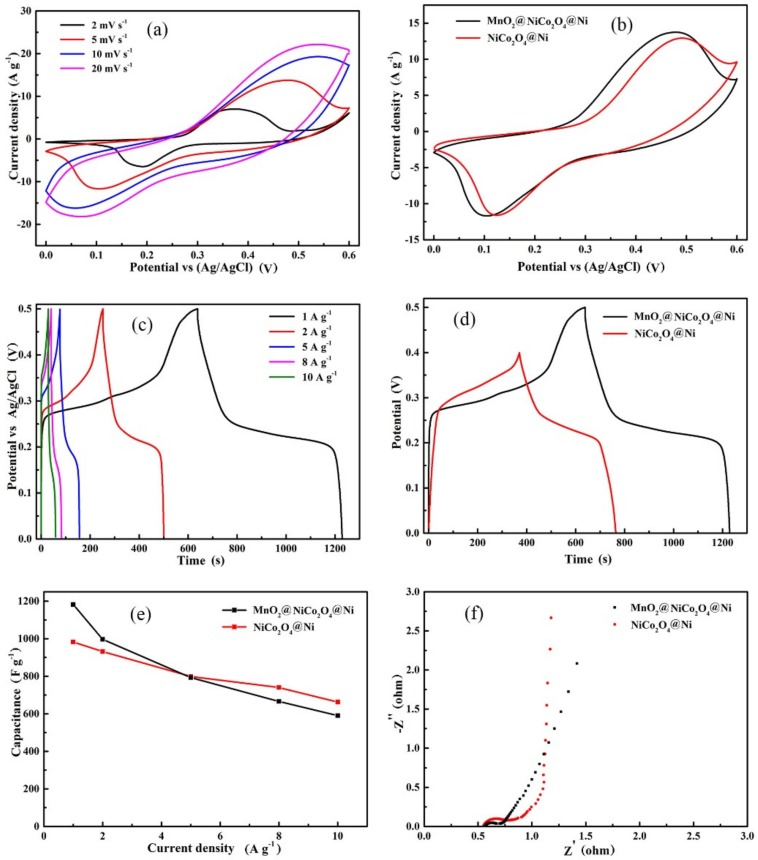
(**a**) Cyclic voltammetry (CV) curves of MnO_2_@NiCo_2_O_4_@Ni under various scan rates, (**b**) CV curves of MnO_2_@NiCo_2_O_4_@Ni and NiCo_2_O_4_@Ni electrodes at 5 mV·S^−1^, (**c**) galvanostatic charge/discharge (GCD) curves of MnO_2_@NiCo_2_O_4_@Ni under different current densities, (**d**) GCD curves at 1 A·g^−1^, (**e**) dependence of specific capacitance on current density and (**f**) Nyquist plots for of MnO_2_@NiCo_2_O_4_@Ni and NiCo_2_O_4_@Ni.

The electrochemical performances of NiCo_2_O_4_@Ni and MnO_2_@NiCo_2_O_4_@Ni were evaluated. They were measured as electrodes directly in a standard three-electrode configuration in KOH solution. Figure 6a shows the cyclic voltammetry (CV) curves of MnO_2_@NiCo_2_O_4_@Ni over a voltage window of 0.6 V (from 0 V to 0.6 V vs Ag/AgCl) under the scan rates ranging from 2 to 20 mV·s^−1^. There is a pair of reaction peaks, showing its battery-like behavior deriving from Faradaic reactions on the surface of TMOs. A straightforward comparison of CV curves of MnO_2_@NiCo_2_O_4_@Ni and NiCo_2_O_4_@Ni at 5 mV·s^−1^ is shown in Figure 6b. From the CV curves, we can see that they have similar electrochemical performances, but the enclosed area of the NiCo_2_O_4_@Ni electrode is slightly lower than that of MnO_2_@NiCo_2_O_4_@Ni, indicating its lower specific capacitance. The galvanostatic charge/discharge (GCD) curves of MnO_2_@NiCo_2_O_4_@Ni with various current densities are presented in Figure 6c, each curve containing a pair of small plateau regions resulting from redox reactions. The specific capacitances are 1186, 1000, 794, 669 and 596 F·g^−1^ at the current densities of 1, 2, 5, 8 and 10 A·g^−1^, respectively, for MnO_2_@NiCo_2_O_4_@Ni. The specific capacitance for the NiCo_2_O_4_@Ni electrode is decreased from 983 F·g^−1^ at 1 A·g^−1^ to 663 F·g^−1^ at 10 A·g^−1^. The GCD curves of two electrodes at 1 A·g^−1^ are straightforwardly compared and exhibited in Figure 6d. Though they show a similar shape, the MnO_2_@NiCo_2_O_4_@Ni electrode shows a longer charge−discharge time and a higher voltage than the NiCo_2_O_4_@Ni electrode, because it combines two different potential windows of two components, leading to a wider operating voltage than the NiCo_2_O_4_@Ni electrode.

The dependence of specific capacitance on the current density for the two electrodes are summarized and exhibited in Figure 6e. MnO_2_@NiCo_2_O_4_@Ni has a higher specific capacitance than NiCo_2_O_4_@Ni at low current densities, but a lower value at high current densities. This is chiefly attributed to the lower conductivity of MnO_2_ than NiCo_2_O_4_ and its larger mass loading than the NiCo_2_O_4_@Ni electrode. The electrochemical impedance spectra (EIS) of the two electrodes measured by an AC source with a voltage amplitude of 5 mV in the frequency range between 100 kHz and 0.01 Hz are shown in Figure 6f. Each Nyquist plot has a linear part in the low frequency region and a small semicircle in the high frequency region, related to Warburg behavior and kinetic charge transfer resistance, respectively. The equivalent series resistance (Rs) values for the two electrodes are comparable from the intercept of plot and real axis. However, the MnO_2_@NiCo_2_O_4_@Ni electrode exhibits a lower charge transfer resistance and a higher Warburg diffusion resistance than NiCo_2_O_4_@Ni, due to their different configurations and conductivities. However, from the specific capacitance and operation potential, the MnO_2_@NiCo_2_O_4_@Ni electrode exhibits an improved performance.

Then MnO_2_@NiCo_2_O_4_@Ni electrode was selected as a positive electrode and AC@Ni was a negative electrode to build a hybrid SC. The mass loading of AC (ca. 6.3 mg) was balanced before assembly according to charge balance theory [48,49]. The electrochemical performances of the hybrid SC were tested in a two-electrode cell in 2M KOH solution.

**Figure 7:**
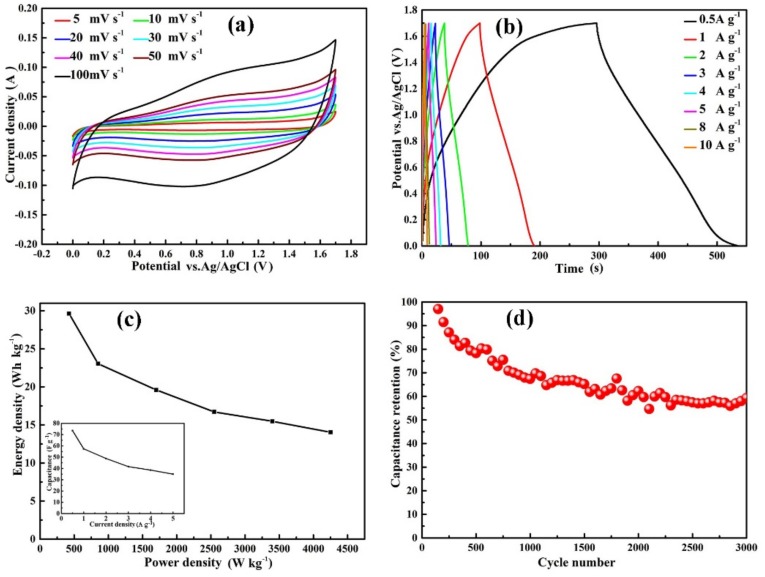
Electrochemical properties of a hybrid supercapacitor (SC) consisting of MnO_2_@NiCo_2_O_4_@Ni//activated carbon (AC), (**a**) CV curves under various scan rates, (**b**) GCD curves under different current densities, (**c**) Ragone plot, inset showing dependence of specific capacitance on current density, and (**d**) stability for 3000 cycles at 2 A·g^−1^.

The CV curves of the hybrid SC under scan speeds from 5 to 100 mV·s^−1^ are shown in Figure 7a. They show the similar shapes with the increasing scan rate with a large potential window of 1.7 V, demonstrating a good reversibility. Each GCD curve of the hybrid SC in Figure 7b exhibits a nearly linear voltage–time relation, showing its capacitive behavior. The specific capacitances of the hybrid SC determined from the discharge curve on the total mass of electrode materials are 73.5, 57.4, 48.8, 38.6 and 35.2 F·g^−1^ at 0.5, 1, 2, 4 and 5 A·g^−1^, respectively. The dependence of specific capacitance for the SC on the current density is shown as an inset in Figure 7c. Less than 50% retention of specific capacitance is achieved with 10-fold increased current density, showing a poor rate capability. A Ragone plot reflects the energy and power density parameters, as exhibited in Figure 7c. It shows that the hybrid SC can deliver the maximum energy density of 29.6 Wh·kg^−1^ at a low power density of 425 W·kg^−1^ under a voltage of 1.7 V. 

The comparison of the MnO_2_@NiCo_2_O_4_@Ni//AC hybrid capacitor in this work to previous reports are listed in Table 1. The energy density of this work is a little lower than previous reports, but the operation voltage is 1.7 V, higher than them [3,28,32,33,35]. The low energy density for this work can be ascribed to the high mass loading of the two electrodes and low surface area of AC. Meanwhile, some ternary composites of MnO_2_@NiCo_2_O_4_ and graphene or carbon nanotubes (CNTs) are involved to ensure high conductivities [33,35]. However, the initial specific capacitance of the hybrid SC loses about 35% after 3000 cycles at 2 A·g^−1^, as exhibited in Figure 7d. Due to its unsatisfactory rate capability and cycling stability, further work is going on to improve them.

## 4. Conclusions

The surface of NiCo_2_O_4_ nanowires grown on Ni foam could be coated with MnO_2_ nanoflakes by electrodeposition. Compared with the conventional chemical method, electrodeposition could be efficiently performed under milder conditions. NiCo_2_O_4_ nanowires were entirely coated with low crystallinity 2D MnO_2_ to generate a core–shell structure. Then, MnO_2_@NiCo_2_O_4_@Ni could be used directly as an electrode for energy storage. It had higher specific capacitance and wider voltage than a NiCo_2_O_4_@Ni electrode. When it was combined with activated carbon to build a hybrid capacitor, the capacitor delivered a high energy density of 29.6 Wh·kg^−1^ at 425 W·kg^−1^ in 1.7 V, implying its great potential for energy storage. However, its rate capability and cycling stability should be further improved in the next step.

## Figures and Tables

**Table 1:** Comparison of electrochemical properties of hybrid SCs.

Two Electrodes of Hybrid SCs	Energy Density/Wh·kg^−1^	Power Density/W·kg^−1^	Work Voltage/V	Specific Capacitance/F·g^−1^	Current Density/A·g^−1^	Reference
NiCo_2_O_4_@MnO_2_ nanospheres//AC	26.6	800	1.6	75	1	[3]
MnO_2_@NiCo_2_O_4_ nanowires//AC	35	163	1.5	112	0.83	[28]
MnO_2_@NiCo_2_O_4_ nanosheet networks//AC	37.5	187.5	1.5	120.9	0.25	[32]
MnO_2_@NiCo_2_O_4_ on graphene//CNTs and graphene	55.1	187.5	1.5	146.2	0.5	[33]
MnO_2_@NiCo_2_O_4_ on graphene//activated graphene	27.8	400.3	1.6	78.1	0.5	[35]
MnO_2_@NiCo_2_O_4_ nanowires//AC	29.6	425	1.7	73.5	0.5	This work
